# Structural Analysis of Treatment Cycles Representing Transitions between Nursing Organizational Units Inferred from Diabetes

**DOI:** 10.1371/journal.pone.0127152

**Published:** 2015-06-01

**Authors:** Matthias Dehmer, Zeyneb Kurt, Frank Emmert-Streib, Christa Them, Eva Schulc, Sabine Hofer

**Affiliations:** 1 Department of Computer Science, Universität der Bundeswehr München, Werner-Heisenberg-Weg 39, 85577 Neubiberg, Germany; 2 Department of Biomedical Computer Science and Mechatronics, UMIT, Eduard Wallnoefer Zentrum 1, A-6060, Hall in Tyrol, Austria; 3 Department of Computer Engineering, Yildiz Technical University, Davutpasa Campus, 34220, Esenler, Istanbul, Turkey; 4 Tampere University of Technology, Korkeakoulunkatu 1, FI-33720 Tampere, Finland; 5 Department of Nursing Science and Gerontology, Institute for Nursing Science, UMIT, Hall in Tyrol, Austria; 6 Department of Pediatrics, Medical University of Innsbruck, Anichstrasse 35, A-6020 Innsbruck, Austria; Nankai University, CHINA

## Abstract

In this paper, we investigate treatment cycles inferred from diabetes data by means of graph theory. We define the term treatment cycles graph-theoretically and perform a descriptive as well as quantitative analysis thereof. Also, we interpret our findings in terms of nursing and clinical management.

## Introduction

The exploration of dynamical systems in nursing and complexity of nonlinear interrelationships of nursing processes has been intricate [1–4]. By studying the scientific literature, we see that there has only been very little use of methods for investigating dynamical relationships in nursing. By mentioning dynamical systems or nonlinear interrelationships of nursing processes, we refer to identify and examine networks [5, 6] for investigating the (topological) structure of the underlying system. Generally speaking, the aim of this paper is to demonstrate the power and usefulness of network-based approaches in nursing. This study has been inspired by recent work due to Dehmer et al. [7] where they employed quantitative graph theory [8] for solving network-based problems in nursing and coined the term *Network Nursing*[7]. In particular, we here infer relational structures (called networks or graphs) reflecting complex relationships between nursing related items. More precisely we generate the networks from spreadsheet data that contains information on patients who were diagnosed with diabetes and underwent an indoor treatment procedure at the Department of Paediatrics at the University Hospital Innsbruck. Our aim is to infer and investigate so-called *treatment cycles* that represent transitions between nursing organizational units of this hospital. In the course of the study, we formalize this problem and then interpret our findings in the context of nursing. We emphasize that reconstructing treatment cycles and presenting the paths patients with homogeneous diagnosis have to went through within departments and hospitals may give important information on facility and capacity needs for both, infrastructural and human resources. Analysis of treatment paths allow a better calculation of needs and one might expect that established treatment cycles lead to optimization of facility, time and human resources.

As already mentioned, there has been only very little work to tackle problems in nursing by means of graph theory. To study this related work, see [7] and section ‘Related Work in Network Nursing’. Here we employ methods from quantitative graph theory [8] to map the inferred treatment cycles to real numbers. This relates to measure their structural complexity. Also, we determine important *patterns* of the treatment cycles to get deeper insights on diabetes treatment processes. The relational structure of a treatment cycles enables us to study the treatment and transitions between nursing organizational units as processes that represent networks. We also determine whether similar transition patterns exist for different groups of patients. Finally we strongly believe that our findings are highly beneficial for optimization issues performed by nursing and clinical management. This will be underpinned by concrete findings and interpretation (see section ‘Interpretation of Treatment Cycles in The Context of Nursing’).

## Methods and Results

We hereby declare that ethics/IRB approval was not required as all data were anonymized and de-identified before access by the researchers. The data does not contain any patient information.

### Related Work in Network Nursing

In recent work of Dehmer et al. [7], they coined the term Network Nursing as synonym for employing quantitative graph theory [8] in nursing science. That means, one uses quantitative techniques to characterize or compare networks inferred from nursing-relevant data sets. Examples can be found in [7]. We briefly repeat the two major directions of quantitative graph theory [8]. (i) Graph characterization relates to characterize a network based on so-called graph invariants. A graph invariant is a measure of a graph that gives equal values for isomorphic networks [9]. (ii) Graph comparison deals with measuring the structural similarity/distance between networks by using quantitative measures, see [10–12]. More recent related work on this subject in relation to topological graph measures can be found in [13–15].

In summary we demonstrated in [7] that quantitative graph theory can be used for analyzing nonlinear interrelationships of nursing processes and, in particular, for their mathematical description, analysis and visualization.

Also we already found [7] that there has only been very little work towards network-based approaches for analyzing scientific problems in nursing-related areas, see [7]. For discussing the state of the art of this area, we refer to [7] but here extend our literature review by elaborating on contributions for using social network analysis in nursing science.

An important network-based topic in nursing science has been to explore communication networks by employing techniques from social network analysis [16–20]. An example thereof are networks representing social interactions between nursing staff. Consequently, several network measures used in social network analysis such as density and centrality have been used to investigate this class of social networks in the context of nursing, see [17, 19]. Cott [21] analyzed communication patterns of interdisciplinary care teams inferred from communication networks [21].

A particular goal of this study was to generate a hierarchy of the nursing staff by means of networks. Benham-Hutchins [16] performed social network analysis to investigate communication processes among the care employees during the transportation process of patients. The study revealed that transportation was not performed by a professional team; they found that there is an individual and unique staff interaction. Similarly, Effken [17] explored relationships between transportations of patients among different care units and patient safety and quality outcomes by using communication networks. To do so, several known network measures have been utilized to perform the analysis of the communication networks. As a result, the network measures indicated some correlation between the staff communication and safety and quality outcome.

Multi-dimensional queuing problems such as assigning patients to nursing staff or assigning rooms to patients efficiently in an organisation with limited sources have been also tackled by using networks [22, 23]. For example, Hershey et al. [22] employed queuing networks to model and predict the capacity change and the patient flow in a health unit that has limited sources. A hypothesis to be discussed in [22] was that the proposed technique may find the proper solution to maximize the service quality with a limited cost and current capacity. Later, Dowsland and Thompson [23] developed modern heuristics to solve nursing scheduling problems efficiently. Merrill et al. [24] investigated communication networks of 11 different local health organizations to identify common and different organisational features of these health organizations. These networks have been analyzed by using several graph measures. A hypothesis of [24] was whether the utilized network parameters may be used to decide about organizational issues such as communication, integration, and source allocation, see [24]. Similar studies have been formed in [25]. There, communication networks representing interactions among Local Health Organizations (LHO) have been constructed and analyzed by utilizing methods from structural network analysis and statistics.

### Disease and Data

The data we have used inferred from type 1 diabetes was available as spreadsheet. We emphasize that no personal patient data is involved as we perform a mathematical analysis. To start, we give some facts about this disease.

Although several types of diabetes are known and classified in childhood, type 1 diabetes is the most common type, characterized as insulin deficiency and the necessity to frequent blood glucose measurements and insulin injection several times a day. Childhood diabetes is a chronic disease with its onset at any age from 1 year to adolescence. The numbers are increasing worldwide for unknown reasons [26] in Austria the increase is about 5% per year [27]. Due to this significant increase adoption of facilities is required. Diagnosis of type 1 diabetes is made in acute conditions (emergency ward) and a huge proportion of patients are in a very severe physical condition, so called ketoacidosis. The diagnosis is followed by several treatment steps; therefore numerous facilities within the hospital are needed. Emergency room, laboratory facilities, intensive care unit, ward and the teaching centre are involved in diagnosis and treatment of patients with diabetes. To analyse how often these facilities are used in our hospital and how the connection of these facilities influences the transfer of patients, we retrospectively collected data from all patients diagnosed with diabetes at the Department of Pediatrics, University Hospital of Innsbruck from 2005 until 2013. The diagnosis diabetes was defined by the international statistical classification of disease and related health problems (ICD 10 code) E 10.-, which classifies the diagnoses diabetes. We did not differentiate between different types of diabetes, but can state, that more than 90% of our patients were classified as type 1. Further differentiation would not have any influence on the pathway the patient had to go through within the hospital. We therefore focused on diabetes as relatively homogenous diagnoses. Only patients seen at the Department of Pediatrics were included in the analysis, adult patients treated at the adult diabetes services were excluded.

### Generation of Treatment Cycles

According to the definition of a *nursing network*[7], we conclude that the treatment cycles inferred from our data sets (see section ‘Disease and Data’) belong to this class of networks. To start, we first explain some basic characteristics of the data set which was available as spreadsheet.

We note that each patient may have several application records for different health cases. Also, more than one nursing diagnosis can be assigned for a particular case belonging to a patient. From this it follows that each patient possesses several networks as different cases can be assigned to the patient. Graph-theoretically speaking [5, 7], a graph is a tuple *G* = (*V*, *E*) where ∣*V*∣ < ∞ is its vertex set and *E* its edge set, respectively. In our study we only consider directed graphs, i.e., *E* ⊆ *V* × *V*. ∣*V*∣ is the number of vertices of *G* and *E* is the number of edges of *G*. ∣*V*∣ is often referred to as order of *G*. In this light, the nursing terms represent nursing organizational units belonging to the same case for a given patient; they are the vertices *v*_1_, *v*_2_, …, *v*_∣*V*∣_ of the resulting nursing network. An edge (*v*_*i*_, *v*_*j*_) is a transition from the nursing organizational unit *v*_*i*_ ∈ *V* to *v*_*j*_ ∈ *V*. It may be interpreted as a relocation from unit *v*_*i*_ ∈ *V* to *v*_*j*_ ∈ *V*.

As we also need to consider edge and vertex weights, we end up with graphs like

$$\begin{array}{c} G=(V,E,f_{E},f_{V},A_{V},A_{E}). \end{array}$$ (1)

In order to explain this notation, we define edge and vertex labeling functions *f*_*E*_:*E* → *A*_*E*_ and *A*_*E*_ ⊂ ℕ. *A*_*E*_ is called the edge alphabet of *G*. Also, we define *f*_*V*_:*V* → *A*_*V*_ where *A*_*V*_ ⊂ *A* ^⋆^ and *A* is an alphabet. *A*_*V*_ is the vertex alphabet of *G*. Here we see that the edge weights are natural numbers defined by the absolute difference of the differences of outgoing date and acquisition date in the hospital of the target and start vertex (nursing terms) plus 1. The edge weight is expressed by days which are obviously natural numbers. The vertex labels represent words *w* ∈ *A*_*V*_ over an alphabet *A* where *w* ∈ *A* ^⋆^.

By using these preliminaries, we are able to state an important definition.

**Definition 1** *A treatment cycle is a directed graph G = (V, E, f_E_, f_V_, A_V_, A_E_) (see* Eq 1 *). The vertices v ∈ V are nursing organizational units. The edges e = (v_i_, v_j_) are transitions (relocations) from a nursing organizational unit to another. The vertex alphabet A_V_ contains the names of the nursing organizational units. The edge alphabet A_E_ contains the weights. Here these weights are time differences between access and departure time.*

**Fig 1:**
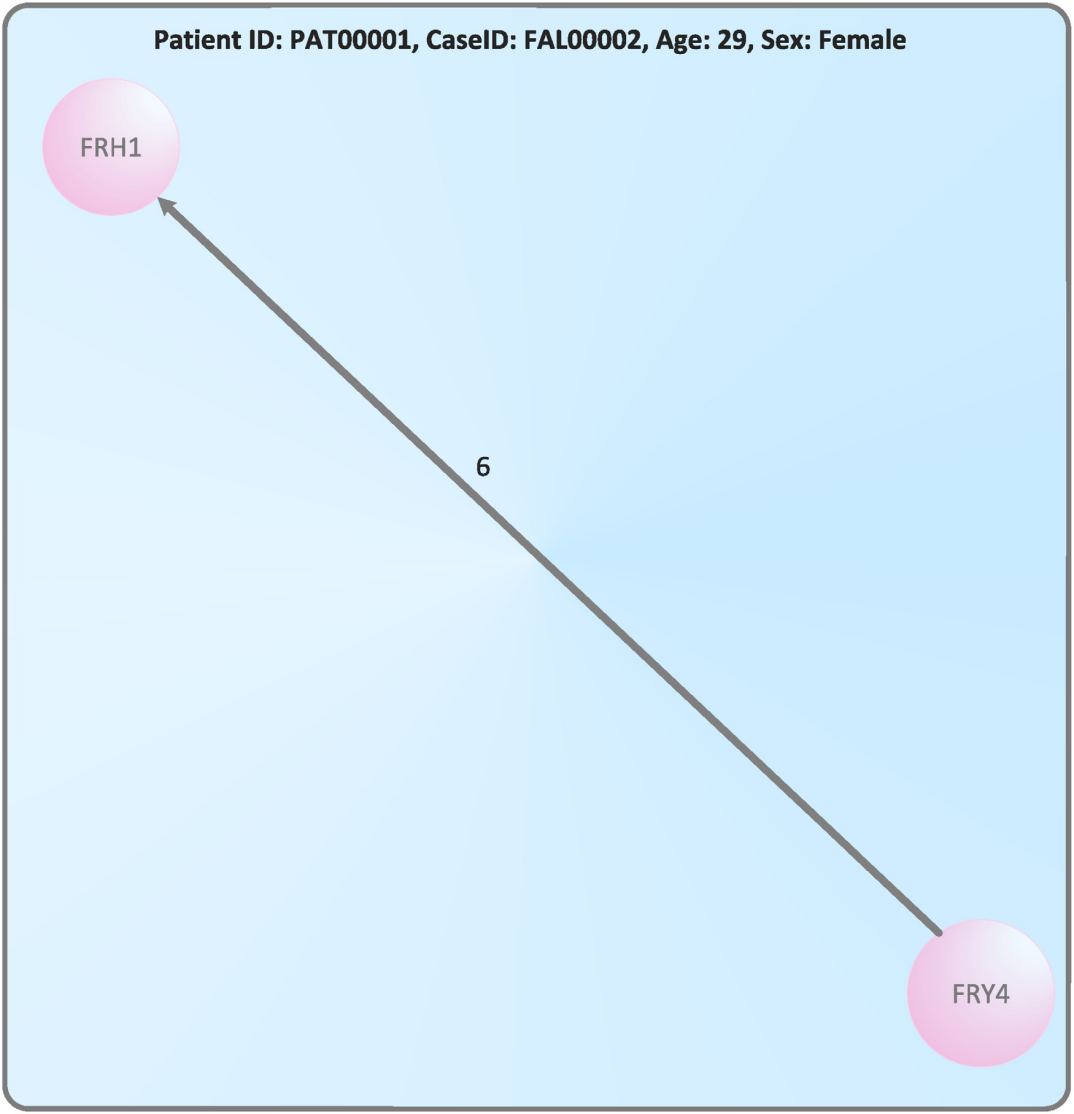
The network *G* = (*V*, *E*, *f*_*E*_, *f*_*V*_) for a patient (ID PAT00001) where *V* = {*v*_1_, *v*_2_}, *f*_*V*_(*v*_1_) = FRY4, *f*_*V*_(*v*_2_) = FRH1, *T* = {FRY4, FRH1}, *E* = {*e*_1_}, and *f*_*E*_(*e*_1_) = 6.

**Example 1** *We explain the nursing network generation by way of example. For this, we consider* Table 1. *We see that there exists a patient with patient ID PAT00001 and case ID FAL00002. These two entries have the nursing terms (nursing organizational units) FRY4 and FRH1 representing the vertices of the treatment cycle G (see* Eq 1 *). It holds f_V_(v_1_) = FRY4 and f_V_(v_2_) = FRH1. (FRY4, FRH1) is the only transition (relocation) between these two units. Finally the treatment cycle for this case ID of the patient has only 2 vertices. Its edge label is f_E_(e_1_) = 6 and ∣V∣ = 2, ∣E∣ = 1. Here we obtain the value of f_E_(e_1_) by first calculating the difference of the outgoing and acquisition dates for the vertex FRY4 which is 1 day. Further, the difference of the outgoing and acquisition dates for the vertex FRH1 is 6 days. Then we yield 6-1+1 = 6. The resulting network is shown by* Fig 1.

**Table 1:** Example entries for a case of a patient with the ID PAT00001.

Patient ID	Case	Nursing term	Acquisition Date OE	Outgoing Date pfl. OE
PAT00001	FAL00002	FRY4	03.11.2005	04.11.2005
PAT00001	FAL00002	FRH1	04.11.2005	10.11.2005

**Fig 2:**
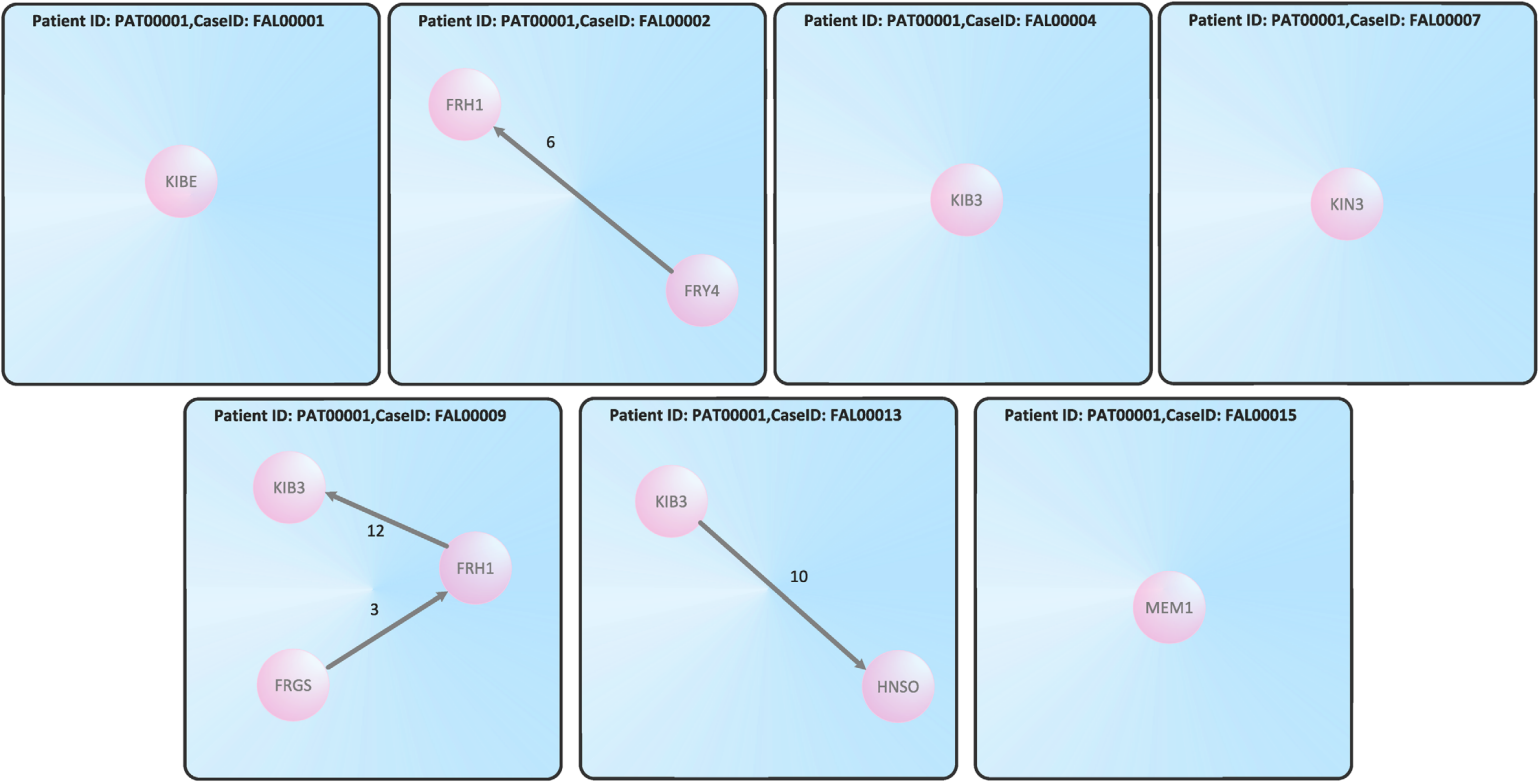
Individual treatment cycles for patient ID PAT00001.

Now we turn to the problem that a particular patient may possess more than one treatment cycles. Then these graphs are inferred from all cases of the patient. To illustrate this process, we examine all cases of the patient PAT00001; this patient has 7 different cases, see Table 2. Thus, this results in 7 individual nursing networks for patient PAT00001, see Fig 2. Also, the case IDs and the vertices *v* ∈ *V* of the resulting graph *G* = (*V*, *E*, *f*_*E*_, *f*_*V*_, *A*_*V*_, *A*_*E*_) for each case are shown by Table 2.

**Table 2:** Different cases for patient PAT00001 and the elements of the vertex alphabet.

**Case No**	**Case ID**	**Nursing terms (Vertex Names)**
1	FAL00001	KIBE
2	FAL00002	FRH1, FRY4
3	FAL00004	KIB3
4	FAL00007	KIN3
5	FAL00009	FRGS, FRH1, KIB3
6	FAL00013	HNSO, KIB3
7	FAL00015	MEM1

**Example 2** *Based on* Table 2, *we see that for case FAL00001 the resulting treatment cycle is an empty graph. In particular, we yield G = (V, E, f_E_, f_V_, A_V_, A_E_) and V = {v_1_}, A_V_ = {KIBE}, f_V_(v_1_) = KIBE, E = {} and A_E_ = ∅. It holds ∣V∣ = 1 and ∣E∣ = 0. See* Fig 2.

**Example 3** *Equally we conclude by considering* Table 2 *that for case FAL00009 the resulting treatment cycle is represented by* Eq 1 *, where V = {v_1_, v_2_, v_3_}, A_V_ = {KIB3, FRH1, FRGS}, f_V_(v_1_) = KIB3, f_V_(v_2_) = FRH1, f_V_(v_3_) = FRGS E = {e_1_, e_2_} and A_E_ = {3,12} ⊂ ℕ. It holds ∣V∣ = 3 and ∣E∣ = 2. See* Fig 2.

**Fig 3:**
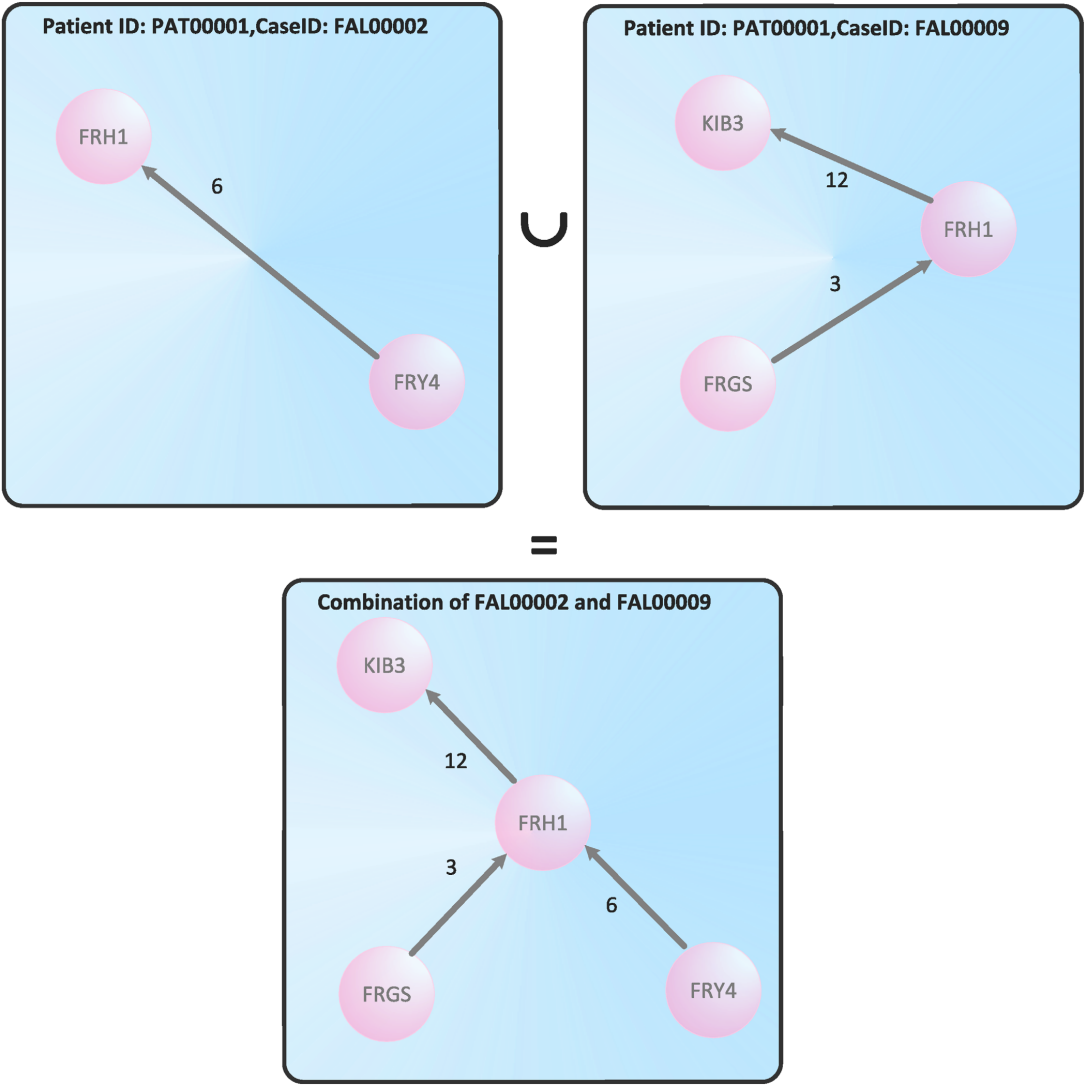
The union of the treatment cycles for the cases FAL00002 and FAL00009.

We mention that it is straightforward to construct a joined treatment cycle as the graph-theoretical union of several graphs for a patient that contains all nursing terms (nursing organizational units) belonging to different cases. We demonstrate this by way of example for the cases FAL00002 and FAL00009, see Fig 3.

### Structural Analysis of Treatment Cycles

In this section, we perform a structural analysis of the inferred treatment cycles. In order to start, we first examine the size of the networks we are dealing with. Then, we explore whether the networks are connected, that means they consist of one component only and, therefore, they do not possess isolated vertices. Note that the available spreadsheet has 1528 entries for 298 patients. From this, 1211 networks have been generated for different cases of patients. Also we generated a joined network by performing the graph-theoretical unification of several networks for each patient. As a result, 298 *joined treatment cycles* have been generated. In total, 1509 treatment cycles have been obtained. Table 3 show the different treatment cycles defined and classified. We observe that the majority of the treatment cycles represent empty graphs, i.e., *E* = {}. Only for 155 treatment cycles we obtain ∣*V*∣ > 1. Also we see that 76 networks out of 298 joined treatment cycles have the property ∣*V*∣ > 1. This is the reason we have eliminated most of the networks.

**Table 3:** Number of the connected and disconnected treatment cycles inferred from the data.

	**Treatment Cycles of individual cases**	**Joined treatment cycles**
# treatment cycles	1211	298
# treatment cycles with ∣*V*∣ > 1	155	76
# connected treatment cycles	155	24
# disconnected treatment cycles	-	52

Special network topologies we have observed when generating the treatment cycles are linear and cyclic graphs. As already mentioned, all treatment cycles are directed, i.e., *E* ⊆ *V* × *V*. A cyclic graph contains a path like *v*_0_, *v*_1_, …, *v*_0_. That means the start and the end vertex of this path are equal. Linear graphs are by definition acyclic (i.e., the number of cycles equals zero). They possess a start vertex *v*_1_ ∈ *V* and an end vertex *v*_∣*V*∣_ ∈ *V*. *v*_1_ has one outgoing edge and *v*_∣*V*∣_ has one incoming edge. The intermediate vertices *v*_2_, …, *v*_∣*V*∣−1_ have one incoming and outgoing edge, respectively.

**Fig 4:**
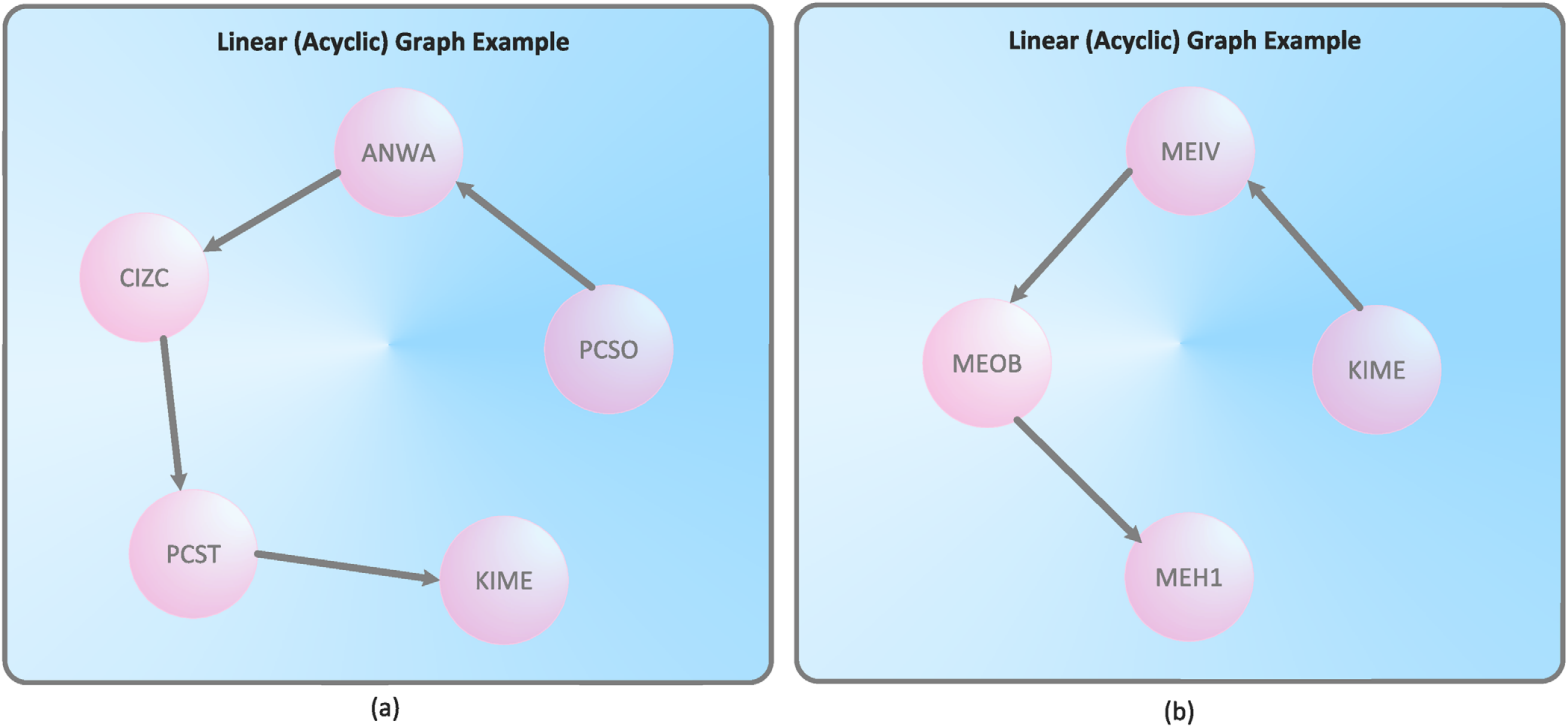
Examples of the linear (acyclic) treatment cycles.

**Fig 5:**
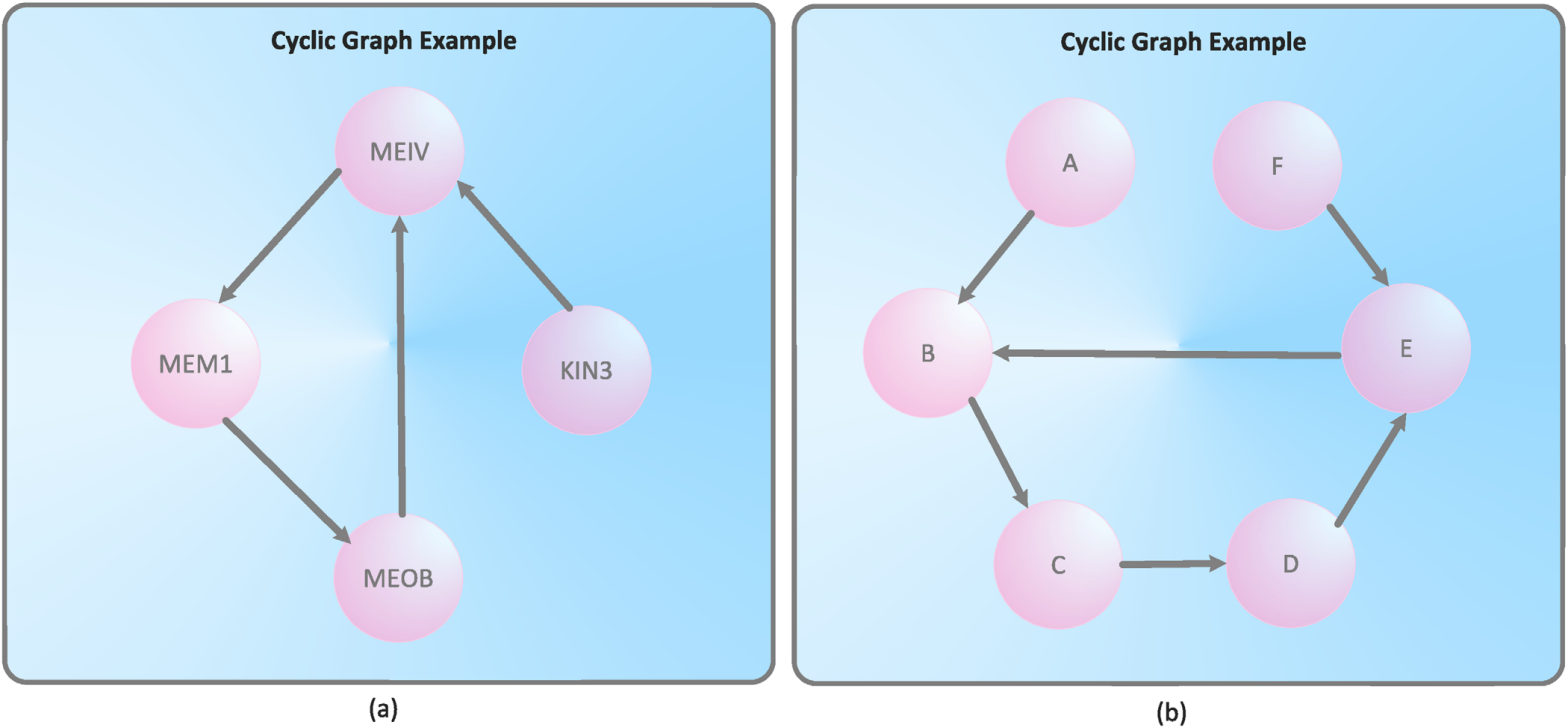
Examples of the cyclic treatment cycles.

Table 4 summarizes the result of the structural analysis we have performed. We see straightforwardly that most of the treatment cycles are linear graphs with ∣*V*∣ = 2. That means the particular patient has only one relocation during the treatment. 21 graphs are linear chains with ∣*V*∣ > 2. An example can be seen in Fig 4. The patients assigned to these nursing networks have more than one relocation in the course of the treatment. Interestingly, we only found 5 networks containing cycles, see Fig 5. These networks contain treatment cycles that start and terminate on the same nursing organizational unit (representing vertices of the nursing network) at Medical University Innsbruck. In summary, we conclude that the majority of patients have had very few relocations in the course of treatment. Most of these patients only needed a single relocation during a particular case. As a conclusive remark, we interpret these results from a nursing-related point of view in the section ‘Interpretation in The Context of Nursing’ and draw several conclusions.

**Table 4:** Number of linear and cyclic treatment cycles inferred from the data.

**Network features**	**# Treatment Cycles**	**# Joined treatment Cycles**
# linear treatment cycles with ∣*V*∣ = 2	129	16
# linear treatment cycles vertex number larger than 2	21	7
# cyclic treatment cycles	5	1

#### Structural Analysis of Treatment Cycles by Using Structural Indices

Apart from the analysis we have performed in the previous section, we also apply structural graph measures [12, 28, 29] to the inferred treatment cycles. A structural graph measure (index) *I* is a mapping *I*:𝓖 → ℝ. 𝓖 is a class of graphs. We see that a structural index maps graphs to the real numbers; such an index can be interpreted as a complexity measure [30] for determining the structural complexity of graphs.

In the following we apply two concrete structural graph measures to the inferred treatment cycles. We start with the well-known Wiener index that has been defined by [31]

$$\begin{array}{c} W\left(G\right)=\frac{1}{2}\sum_{i=1}^{\left|V\right|}\sum_{j=1}^{\left|V\right|}d(v_{i},v_{j}). \end{array}$$ (2)

*d*(*v*_*i*_, *v*_*j*_) is the length of the shortest path between the vertices *v*_*i*_, *v*_*j*_ ∈ *V*. We see that the Wiener index is the sum of all distances between the vertices of a treatment cycle *G*. We emphasize that recent work on the Wiener index showing the richness of the concept of distance-based indices can be found in [32, 33]. The second index we introduce is the Randić index [34, 35]

$$\begin{array}{c} R\left(G\right)=\sum_{(v_{i},v_{j})\in E}{\left[k_{v_{i}}\;k_{v_{j}}\;\right]}^{-\frac{1}{2}}. \end{array}$$ (3)

*k*_*v*_*i*__ is the degree of the vertex *v*_*i*_, i.e., the number of edges incident with this vertex. We see that Eq 3 has been defined for undirected networks *G*. In order to define the Randić index for directed treatment cycles, we apply Eq 3 to the in-degrees [5] and out-degree [5] of the graph correspondingly and define

$$\begin{array}{c} R{\left(G\right)}_{fin}=\frac{R{\left(G\right)}_{in}+R{\left(G\right)}_{out}}{2}. \end{array}$$ (4)

**Fig 6:**
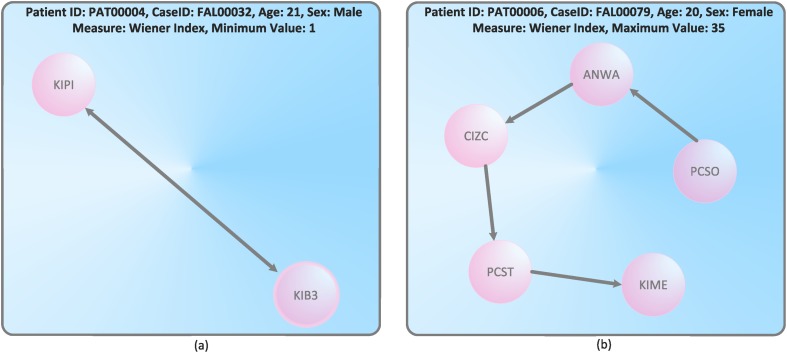
Treatment cycles with the minimal and maximal Wiener index.

**Fig 7:**
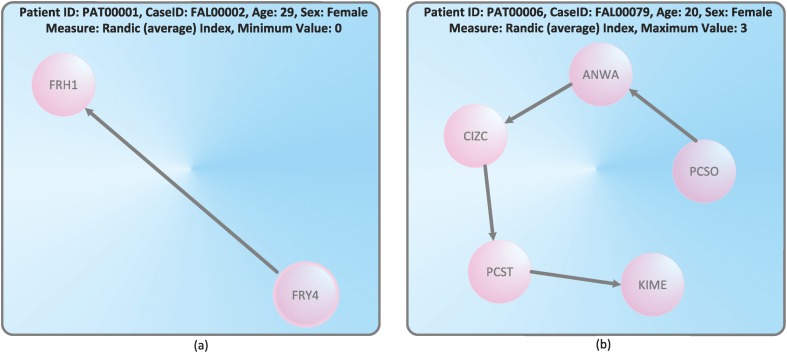
Treatment cycles with the minimal and maximal Randić index.

This tries to identify patients with extremely low number of connectivity and patients with multiple and complex transfers resulting in a high number of connective points, see Figs 6 and 7. We start by considering Fig 6(a) and see that the graph with minimal Wiener index has linear structure. By using the fact that the Wiener index has often been used to detect *structural branching*[36], the obtained result seem to be plausible. The treatment cycle with linear structure is obviously minimally branched. Fig 6(b) shows also a linear treatment cycle that is maximally branched. If we define the left graph as $G_{l}^{W}=(V_{l}^{W},E_{l}^{W})$ and the right graph as $G_{r}^{W}=(V_{r}^{W},E_{r}^{W})$, the just obtained result is obvious as $|V_{l}^{W}|<|V_{r}^{W}|$.

A similar discussion of the results shown by Fig 7 follows. By knowing that the Randić index is a classical *connectivity index*[29], the obtained results seem to be plausible too. The graph depicted by Fig 7(a) is the treatment cycle $G_{l}^{R}=(V_{l}^{R},E_{l}^{R})$ with minimal connectivity. The treatment cycle $G_{r}^{R}=(V_{r}^{R},E_{r}^{R})$ shown by Fig 7(b) has maximal connectivity. Again, the result is obvious because $|V_{l}^{R}|<|V_{r}^{R}|$.

### Interpretation of Treatment Cycles in The Context of Nursing

Also we analyzed the frequencies in which the nursing terms representing nursing organizational units occur. We performed this analysis for cyclic treatment cycles and consider the results in Table 5. We see that the depicted values satisfy # > 1. The nursing organizational unit KIB1 (Department of Pediatrics 1) occurs most in the treatment cycles. The terms KIB3 (Department of Pediatrics 3), KIB4 (Department of Peditrics 2), and KIPI (pediatric intensive care unit) occur two times, respectively.

**Table 5:** Total frequencies of the most common nursing terms (nursing organizational unit) which occur in cyclic treatment cycles.

**Nursing organizational unit**	**Frequency (#)**
KIB1	4
KIB3	2
KIB4	2
KIPI	2

As mentioned above, a treatment cycle is a graph reflecting the transitions between nursing organizational units which are its vertices. An important question for nursing management is to analyse how often patients need to be moved between wards and facilities. This process is here defined as *transfer*. We note that in graph-theoretical terms transfer means transition (see Section ‘Introduction’ and Section ‘Generation of Treatment Cycles’) as it describes patients moved from one facility to another. The transfer of patients between facilities is a time intense procedure including prearrangements, the transfer itself and handing over of information, treatment and data of the patient. Beside time, human resources are bounded with transfers. Deeper insights on this specific area may give useful information for optimization of procedures and nursing management.

In our setting a high proportion of patients have only one contact point (vertex). In graph-theoretical terms, they represent empty graphs. This finding suggests, that patients with this specific diagnosis (ICD 10 E10.- representing diabetes) are mainly seen in one specific facility. This allows the development of competence in this facility in terms of structural competence as well as personnel competence. Nursing strategies can be developed as patients with the same/similar needs repeatedly appear in this facility. In regard to quality improvement this is a very important finding.

In the hospital setting analyzed in our study we furthermore observed that the most frequent transfer occurred between intensive care unit (KIPI) and ward (KIBE, KIB1, KIB3 and KIB2).

This transfer model is not surprising, as a certain number of patients with diabetes show a severe ketoacidosis which indicates intensive care treatment. After stabilization at the intensive care unit a transfer to the diabetes ward is possible and necessary to introduce subcutaneous insulin treatment and diabetes educational programs.

In summary we investigated a homogenous patient cohort with several types of diabetes, mainly type 1 diabetes, and could identify treatment cycles which occurred in regular repetition. Furthermore we could show that patients with the same or similar diagnosis were basically treated at the same ward which implicates, that the same team of doctors and nurses is taking care of these patients. This is important knowledge, as this setting allows the development of expertise in diagnosis, treatment and education of this specific patient group. The expertise of a team treating patients with a group of diagnoses is important not only because of improvement in health outcome and prognosis. This expertise is also necessary to improve efficiency and time management. Therefore we can conclude, that the simple treatment cycles which we have observed affirm that this hospital has already optimized patient care by transferring patients diagnosed with diabetes at one specifically prepared and educated ward. This study gives an example how the analysis of treatment cycles in hospitals may be a helpful tool to identify efficient working loops but also allows recognizing areas which possible could improve from structural changes.

Formally, the results of our findings are summarized in Table 6. We observe that the transfer (KIPI, KIBE) is the most frequent one. The second and the third ranked transitions among the observed ones are (KIPI, KIB1) and (KIPI, KII2), respectively. Also this study reveals that nursing organizational unit KIPI is the most important one as it occurs in the graph-theoretical relation (KIPI, *v*_*j*_), *v*_*j*_ ∈ *V* most.

**Table 6:** Most frequently observed transitions between nursing organizational units.

Frequency (#) Ranking	**#**	Transitions **(*v*_*i*_, *v*_*j*_)**
1	36	(KIPI, KIBE)
2	25	(KIPI, KIB1)
3	10	(KIPI, KII2)

## Summary and Conclusion

In this paper we dealt with exploring so-called treatment cycles by using graph theory. We analyzed these graphs descriptively and quantitatively. To perform quantitative graph theory [8], we used topological graph measures such as the Wiener and Randić index [35]. To the best of our knowledge, we defined a directed version of the Randić index for the first time. This is required as the treatment cycles represent directed graphs.

We think that our approach offers an interesting theoretical approach for some important practical problems in the wider context of nursing. As such, it would be interesting to explore connections toward personalized medicine and its clinical realization. Given the fact that in a genomics context the network representation of high-throughput data, e.g., in form of gene regulatory networks, is a widely used approach in network medicine [37] there should be a natural interface allowing the integration of the heterogeneous data types.

From the view of Nursing Science, the structural analysis of treatment cycles for children suffering from Diabetes Type 1 will break new ground in science. Whether the performed analysis is confirmed also in nursing practice demands further research. It is planned, by means of a comparative approach, to compare treatment cycles for adults with other chronic diseases. But this requires the availability of the data. Additionally, nursing practitioners should assess and analyze by means of qualitative research methods the everyday feasibility of the structural analysis model from an individual and system-critical view.

## Supporting Information

S1 Data csv file containing the spreadsheed data to infer the treatment cycles. (CSV)
